# Activities of Daily Living and Categorization Skills of Elderly with Cognitive Deficit: A Preliminary Study

**DOI:** 10.3390/brainsci11020213

**Published:** 2021-02-10

**Authors:** Dulce Romero-Ayuso, Cristian Cuerda, Carmen Morales, Ricardo Tesoriero, José Matías Triviño-Juárez, Antonio Segura-Fragoso, José A. Gallud

**Affiliations:** 1 Department of Physical Therapy, Occupational Therapy Division, Faculty of Health Sciences, University of Granada, 18016 Granada, Spain; 7carmenms@gmail.com; 2 Computing Systems Department, University of Castilla-La Mancha, 02071 Albacete, Spain; cristian.cuerda@uclm.es (C.C.); ricardo.tesoriero@uclm.es (R.T.); 3 Primary Care Center Zaidín Sur, Granada Metropolitan Sanitary District, 18007 Granada, Spain; jmtjuarez@hotmail.com; 4 Faculty of Health Sciences, University of Castilla-La Mancha, Talavera de la Reina, 45600 Toledo, Spain; Antonio.Segura@uclm.es

**Keywords:** activities of daily living, health information technology, cognitive impairment, cognition, occupational therapy

## Abstract

Cognitive dysfunction affects the performance of Activities of Daily Living (ADL) and the quality of life of people with these deficits and their caregivers. To the knowledge of the authors, to date, there are few studies that focus on knowing the relationship between personal autonomy and deductive reasoning and/or categorization skills, which are necessary for the performance of the ADL. The aim of this study was to explore the relationships between ADL and categorization skills in older people. The study included 51 participants: 31 patients with cognitive impairment and 20 without cognitive impairment. Two tests were administered to assess cognitive functions: (1) the Montreal Cognitive Assessment (MoCA); and (2) the digital version of Riska Object Classification test (ROC-d). In addition, the Routine Tasks Inventory-2 (RTI-2) was applied to determine the level of independence in activities of daily living. People with cognitive impairment performed poorly in categorization tasks with unstructured information (*p* = 0.006). Also, the results found a high correlation between cognitive functioning and the performance of ADLs (Physical ADL: *r* = 0.798; *p* < 0.001; Instrumental ADL: *r* = 0.740; *p* < 0.001), a moderate correlation between Physical ADLs and categorization skills (unstructured ROC-d: *r* = 0.547; *p* < 0.001; structured ROC-d: *r* = 0.586; *p* < 0.001) and Instrumental ADLs and categorization skills in older people (unstructured ROC-d: *r* = 0.510; *p* < 0.001; structured ROC-d: *r* = 0.463; *p* < 0.001). The ROC-d allows the assessment of categorization skills to be quick and easy, facilitating the assessment process by OT, as well as the accuracy of the data obtained.

## 1. Introduction

The main risk factor for the development of cognitive decline is aging [1]. According to the WHO, the prevalence of dementia in the population over 60 years is 5–8% [2]. This represents a burden for patients, families, and countries and is, therefore, a major public health problem that must be addressed [1]. One of the most relevant aspects of cognitive impairment is the loss of personal autonomy and independence in everyday life [2]. Cognitive impairment leads to greater difficulty in performing activities of daily living (ADLs), such as that seen in people with dementia [3], and affects the health-related quality of life of both the patient and the primary caregiver [4]. The loss of independence begins with a decline in the instrumental activities of daily living (IADL) (i.e., shopping, using the media, managing money, among others), which require interaction with the environment, cognitive skills, and social interaction [2]. Although the difficulty to perform IADL is a frequent complaint in older people, the assessment of deficits in IADL regarding cognitive skills has not been widely studied [5,6]. Deficits in executive functioning (cognitive flexibility, organization, initiation, and maintenance of actions or plans), processing speed, and delayed recall of memory tasks have been associated with poorer performance in ADLs [7,8]. This has been related to the frontal aging hypothesis, which suggests that the first cognitive functions to deteriorate are those associated with the functioning of the prefrontal cortex, such as executive functions [6]. Based on Diamond’s cognitive model, reasoning and categorization skills could be understood as an advanced skill of executive functions, together with planning and problem solving, which depend on an adequate functioning of working memory, inhibitory control, and cognitive flexibility [9]. Executive functions can be assessed using measures of categorization and reasoning [7,10]. One study found that people without reasoning deficits had less difficulty performing IADLs [11]. Other studies examined the categorization and performance of ADLs but in stroke patients [12]. The categorization appears to be a good predictor of skills related to IADL in patients with brain injury [10]. However, the relationship between categorization, reasoning, and functioning in daily life has been less studied in elderly people with cognitive impairment [12]. In part, this could be because few of the executive functioning assessment tools in the clinical setting include a detailed assessment of categorization skills [13,14]. The categorization is the human capacity to organize the different perceptual stimuli in clusters at different levels of abstraction, which allows us to store and use the knowledge acquired throughout life, in a fast and effective way [15]. The categorization responds to our mind’s need for “cognitive economy”, the way we categorize and organize information to reduce the amount of information we need to learn, remember, and manipulate, and expand the ability to deal with new situations. The categorization, formation of categories and concepts, are carried out through a process of inductive reasoning [16,17], and allows us to organize our world, in colors, sizes, shapes, by semantic categories or in more complex concepts [15]. Although decreased IADL performance is not a determinant of mild cognitive impairment, some studies indicate that there are deficits in the complex factors of IADL in these patients [4]. In fact, the categorization is a fundamental cognitive process that is used in one way or another in almost all ADLs [10] and also it has been related to cognitive flexibility, which allows adaptation to new situations [12].

The increase in life expectancy—together with sociodemographic, clinical, and functional factors [9]—suggest the importance of studying new methods for the early assessment of cognitive functions for the following reasons: (a) adequately analyze the situation and patient characteristics; and (b) initiate prevention, treatment, and rehabilitation plans with the aim of maintaining the greatest possible functionality, personal autonomy, and quality of life. Cognitive tests are essential for establishing the diagnosis, monitoring progression, and evaluating treatments, with assessments ideally brief, reliable, valid, and reflecting clinically significant changes [18]. Our solution focuses on this first stage of evaluation of cognitive impairments, which is the evaluation of categorization abilities. To do this, we start from a traditional methodology such as Lowenstein Occupational Therapy Cognitive Assessment (LOTCA) [19], Toglia Category Assessment (TCA) [20], or Wisconsin Card Sort Test (WCST) [21,22]. In this study, we have created a digital platform with the aim to assess categorization skills that facilitates the diagnosis and assessment of the patient.

To the knowledge of the authors, studies focused on the relationship between categorization skills in IADL in older patients with and without cognitive deficits have not been found. The present study was designed to expand the existing body of research on the relationship between categorization skills and IADL in older people, through a digital object classification task, and the performance of ADLs in older people with cognitive impairment.

In the light of above, the first hypothesis of this study was that there would be statistically significant differences in the performance of ADLs between the group of elderly with normal cognitive performance and the group with elder people with cognitive impairment. The second hypothesis was that the better performance in categorization skills, the better performance in ADL, particularly IADL.

## 2. Materials and Methods

### 2.1. Design

A cross-sectional descriptive study was conducted, comparing the performance in different cognitive test and ADLs between people with and without cognitive impairment. The sampling procedure was intentional.

### 2.2. Participants

A total of 51 community dwelling adults aged 60 years and above participated in this study. The mean age of the participants was 71.25 (SD = 11.46) years. A total of 68.6% (*n* = 35) were women. The determination of the educational level of each participant was checked according to the Spanish educational system: (1) Illiterate when the person had not completed the first studies or had not completed the primary studies, although he knows how to read, write and sign; (2) Primary School when she/he had completed primary or basic general education; (3) Secondary education when the participant had studied high school or vocational training; and (4) Higher education if the person had studied university or higher education [23]. Regarding to educational level, 27.5% (*n* = 14) were illiterate, 31.4% (*n* = 16) had primary education, 27.5% (*n* = 14) secondary education, and 13.7% (*n* = 7) had higher education. To determine the socio-economic status (SES), mainly derived from the occupation, it was used the classification of the group on Social Determinants of the Spanish Society of Epidemiology from the National Classification of Occupations of 2011 [24]. It was divided into three categories: Level I: Directors, managers, and professionals with university training; Level II: intermediate occupations and self-employed; Level III: Manual workers [24]. Concerning this, 15.7% (*n* = 8) were included in level I, 5.9% (*n* = 3) in level II and 78.4% (*n* = 40) in level III. All participants were administered the Cognitive Evaluation of Montreal (MoCA) to screen for cognitive impairment. Twenty patients with cognitive impairment were recruited from the Day Center Los Tulipanes (Granada, Spain) and 31 people without cognitive deficit were recruited in several Social and Leisure Center. Table 1 describes the characteristics of the sample.

Before the assessment of the participants, approval was obtained from Los Tulipanes Day Center Management to conduct the study and to collect the data. Furthermore, written informed consent was obtained previously from the responsible caregivers and/or the participants. The study was performed according to the guidelines of the Declaration of Helsinki and was approved by the Ethics Committee of the University of Granada (protocol code 661/CEIH/2018 with approval date 25 September 2018). Subsequently, a neuropsychologist administered the cognitive tests and an occupational therapist administered the ADL tests.

### 2.3. Instruments

#### 2.3.1. MoCA

MoCA is a brief cognitive test useful to detect mild cognitive impairment and early dementia [25]. It consists of several subtests that assess several cognitive processes: (1) Executive function and visuospatial ability, which consists of three tests: an adaptation of the Trail Making Test B (1 point), the copy of a geometric cube (1 point) and the copy of the clock test (3 points); (2) Naming (naming of three animals with low familiarity: 3 points); (3) Attention, assessed through a task of direct and inverse digits (2 points), sustained attention (1 point) and a series of subtractions (3 points); (4) Language (repetition of complex sentences (2 points) and phonetic fluency (1 point); (5) Abstraction: with two items of abstract verbal reasoning (2 points); (6) Learning and recall: there are two five-word learning trials that are asked after five minutes (5 points); allows to record the strategies that facilitate recall, for example with a semantic key, multiple choice or if there is free recall; (7) Temporal and spatial orientation (6 points).The administration time is between 7 and 10 min, depending on the cognitive state of the patient [13]. According to the scale and MoCA scores for the Spanish population, adjusted for age and educational level, a score lower than 24 was considered to establish cognitive impairment in older people between 66 and 70 years of age. For patients between 71–75 years old, the cut-off point is established at 22 points. A score of fewer than 21 points suggests mild cognitive impairment in patients between 76 and 80 years of age. A score of fewer than 19 points indicates a cognitive decline in those older than 81 years [13].

The complete assessment, with all the assessment instruments, was performed on the same day, for each patient, following the same protocol and order of assessment with all participants. According to the score obtained in the MoCA [13], participants were included into one group or the other (cognitive impairment; without cognitive impairment).

#### 2.3.2. ROC-d

**Figure 1:**
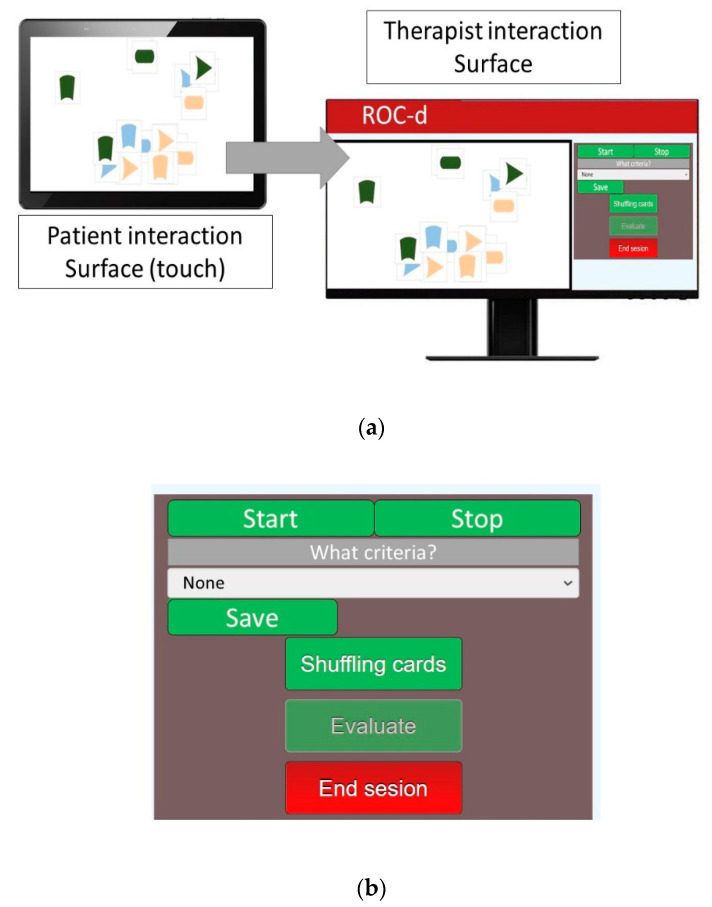
(**a**) ROC-d assessment system distributed user interface; and (**b**) Menu of ROC-d for therapist.

For this study, computer engineers developed a new task based on ROC test. The Riska Object Classification test (ROCUS and ROC) is well established and has been used to assess categorization ability in various populations [26]. This digitized test was named ROC-digital (ROC-d). Similar to the original version [20,27], ROC-d has two subtests: (1) Riska Object Classification Unstructured (ROCUS); and (2) Riska Object Classification Structured (ROC). The items come in 18 shapes in three colors (dark green, light blue, and beige color) and three different shapes (oval, arrowhead, and arched shapes). Of each color there are two pieces with each shape: two ovals, two arrowheads, and two with arched shapes as shown in the Figure 1. ROCUS is unstructured and participants are asked to classify objects into ‘similar groups’, whereas ROC is structured and participants are shown an example of a group before they are asked to classify objects into ‘similar groups’. For each test, performance is rated on a five-point scale, from ‘unable to rate’, ‘able to rate based on one criterion’, ‘able to rate based on two or more criteria after a second attempt’, or ‘able to classify according to two or more criteria’ (Table 2). A touch screen that could be connected to a computer was needed to use the ROC-d, in order to allow the occupational therapist and neuropsychologist to see the patient’s interactions with the touch screen and the activity on the computer.

The procedure for performing the subtest was as follows: 18 flat pieces in 3 colors and 3 different shapes were placed on the patient’s touch screen. The occupational therapist (OT) then asked the patient to organize the pieces into groups that were similar or identical. When the patient finished grouping the pieces, the OT asked about the criteria that had been followed. After noting this, the participant was asked if she/he could propose any other grouping criteria to regroup the pieces. The test had two parts: one in which the pieces were grouped without any pattern and another in which the pattern was followed on the touch screen.

The proposed solution, ROC-d, consists of a client-server application in which the OT can create a personalized session for the patient, including data such as his or her name. Traditionally, the OT provides the patient with a set of cards containing different geometric shapes with different colors, and the patient is asked to group them according to the pattern provided by the OT. Our solution, however, proposes a distributed application, in which the patient works on a touch screen to solve the test, while the OT observes the results in real time on another screen. In this test, as in the original version, the patient is shown a set of cards with geometric figures of different colors.

The system is supported by a distributed user interface (DUI) [28] consisting of two interaction surfaces: patient and OT. The user interface for both interaction surfaces are depicted in Figure 1. While the patient interaction surface enables patients to manipulate shapes on a touch screen display (Figure 1: Patient interaction surface), the OT interaction surfaces enables them to control patient interaction surface (Figure 1: Therapist interaction surface and Menu for ROC-d for therapist).

The control panel enables OT to select the criteria (if required), shuffle cards, report assessment results or end assessment sessions. Besides, it helps OT monitoring cloning patient view to avoid therapy interference in the assessment process in real time. In addition, it also shows session elapsed time. This can be done using the display module of the application results. This module allows, given that a specific session has already been carried out, to visualize which cards have been moved by the patient in a specific time slot, showing their position of origin, the path travelled, and the final position. The OT can modify this time frame to visualize which cards have moved in different lengths of time.

**Figure 2:**
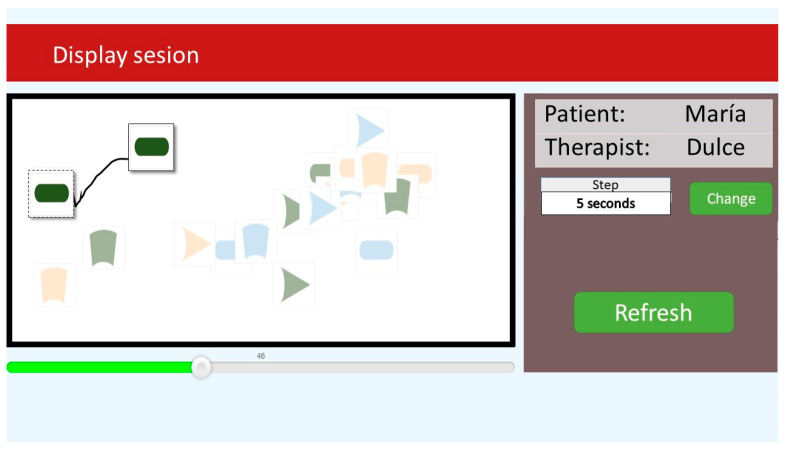
Assessment session summary information.

ROC-d provides therapists with the assessment analysis module. This module enables therapists to review and analyze information of assessment sessions stored into the system. Figure 2 depicts the user interface that summarizes the information to analyze assessment sessions. This user interface is organized into two panels. The right panel shows the list of sessions that OT are able to explore. When they click on a session the information related to the selected session is displayed on the left panel. The assessment session information includes the patient identification, the session starting date and time, the session duration, the assessment results of each ROC-d phase, observations, etc.

**Figure 3:**
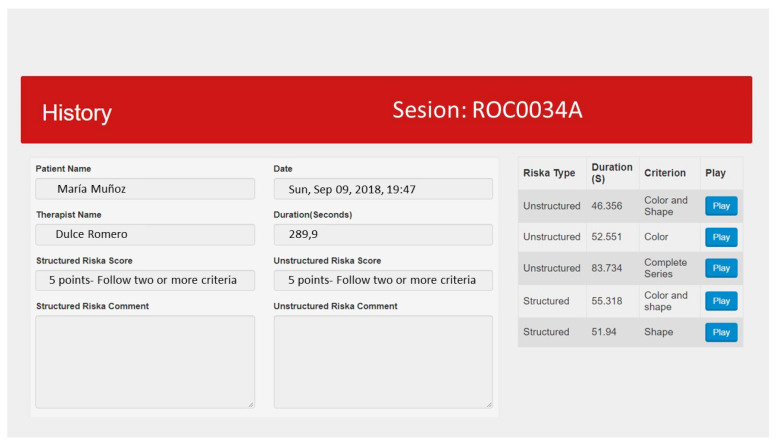
Assessment session review user interface.

The OT is able to review assessment sessions by clicking on the ‘Play’ button next to the session listed in the right panel of Figure 2. As a result, the user interface depicted in Figure 3 is displayed to ‘Browse’ patient actions for a period of time. This user interface displays two panels. The right panel enables OTs to set the period of time to be browsed using the ‘Step’ and ‘Change time’ buttons which define a time period in seconds. The left panel displays the actions performed by the user during the defined period starting at a specific point which is set by the sliding bar displayed at the bottom of this panel. For instance, the OT is able to check patient action from the second 45 to the second 50 of an assessment session by setting the starting time to 45, the step to 5 s, and clicking on ‘Refresh’ button to see the results. These results are displayed on the upper view of the left panel where the set of that was not manipulated by patients are greyed out to highlight those that were moved during the specified period of time. The shapes that were moved are displayed twice where a dashed line square enclosing the shape is used to show the position of the shape at the beginning of the period and a solid line square enclosing the other shape to show the position of the figure at the end of the period. Both shapes are connected through a line showing the path followed by the shape to arrive from the starting position to the end position.

Before administering the digital object classification task (ROC-d), all participants were instructed and asked to complete various test items in a test round to ensure that they understood the task.

#### 2.3.3. Routine Task Inventory-2

The Spanish version of Routine Tasks Inventory 2 (RTI-2) was applied with the aim to know functional status and ADLs performance. The Routine Task Inventory 2 (RTI-2) is a second version of Inventory of Routine Tasks (RTI), an assessment of cognitive abilities in the context of routine daily activities. It allows us to determine the cognitive level of the patient. According to the Allen Model, there are six cognitive levels. The higher the cognitive level, the more independence a person shows in their personal autonomy. It can be completed with three sources of information: patient self-report, a family member or other caregiver’s report, and observations of performance by a therapist. It consists of 14 items such as: personal care, dressing, bathing, walking, feeding, grooming, home establishment and management, meal preparation, financial management, taking medication, cleaning the house, traveling, shopping, and telephoning [29]. The scale of physical activities (basic ADLs) of daily living and IADL were applied; each scale is composed of seven items. Instrumental RTI-2 scale range from 1 to 6, and score for the Physical ADLs scale range from 1 to 5 [29]. The higher the score, the higher the level of independence in performing ADLs. RTI has good psychometric properties, with high inter-rater reliability (*r* = 0.987), test-retest (*r* = 0.906) and good internal reliability (Cronbach alpha = 0.94) [26]. RTI-2 was found to be a valid measure of cognitive impairment [29].

### 2.4. Data Analysis

Descriptive statistics were used for qualitative and quantitative variables. To analyze the differences in qualitative variables between the two groups of comparison (cognitive impairment vs. no cognitive impairment), we used Pearson’s chi-squared test or Fisher’s exact test when the expected values in any of the cells of the contingency table were below five [30]. Spearman’s correlation coefficients were calculated for ADL and categorization skills. The strength of correlation was based on the correlation coefficient (r): negligible (0.0 ≤ 0.3); low (0.3 ≤ 0.5); moderate (0.5 ≤ 0.7); high (0.7 ≤ 0.9); very high (0.9–1.0). In order to analyze the differences in quantitative variables between these two groups, as the distribution of the data were non normal, the Mann–Whitney U test was performed. Age, socioeconomic status, and educational level showed differences between the two groups. As the differences of the scores of ROC-d and RTI-2 between groups were statistically significant, we planned to conduct a multivariate analysis controlling for age, socio-economic status, and educational level as potential confounders. The ADL variables, as continuous and highly asymmetric variables, could not be normalized by the transformation, they were dichotomized by the median. Likewise, the categorical variables were also dichotomized. Finally, once the variables were dichotomized, a multivariate model with binary logistic regression was performed, obtaining the odds ratio (OR) with the 95% confidence interval (95% CI). For the case of a binary logistic regression, with a dichotomous dependent variable, an interpretation of the OR is proposed based on a transformation to Cohen’s d (*d*) [31], as follows: OR ≤ 1.68, the magnitude of the effect is considered ‘insignificant’ (*d* < 0.2); if the OR is >1.68 and <3.47: ‘small’ (0.2 ≤ *d* <0.5); if the OR is ≥3.47 and <6.71: ‘moderate’(0.5 ≤ *d* <0.8), and if the OR is ≥6.71: ‘large’ (*d* ≥ 0.8) [31]. The level of statistical significance was set at *p* < 0.05. The data were analyzed using the IBM SPSS statistical software (version 26.0, IBM Corp., Armonk, NY, USA).

## 3. Results

The differences between the groups of comparison (cognitive impairment and no cognitive impairment) in the bivariate analysis are shown in Table 3. Results showed statistically significant differences in age (*p* < 0.001), educational level (*p* < 0.001) and socioeconomic status (*p* = 0.004) across the two groups. No differences were found in the distribution of gender (*p* = 0.360) between the two groups.

The results of bivariate analysis found significant differences between the two groups in both the score of ROCUS (*p* < 0.001) and ROC (*p* < 0.001). No differences were found in the time of execution of ROC-d (*p* = 0.150). In relation to the performance of ADLs, significant differences were also observed on independence in Physical ADLs (*p* < 0.001) and IADL (*p* < 0.001) (Table 3).

Also, the results showed a significant high correlation between IADL-RTI-2 and Physical ADL-RTI-2 (*r* = 0.818; *p* < 0.001) and IADL-RTI-2 and the MoCA (*r* = 0.740; *p* < 0.001) moderate between IADL-RTI-2 and ROCUS (*r* = 0.510; *p* < 0.001) and IADL-RTI-2 and ROC (*r* = 0.463; *p* = 0.001) (Table 4). In addition, Physical ADL-RTI-2 showed significant correlations between each ROC-d subtest, being moderate with both ROCUS (*r* = 0.547; *p* < 0.001) and ROC (*r* = 0.586; *p* < 0.001). A high correlation between MoCA and Physical ADL-RTI-2 and a moderate one were observed between the two ROC subtests (*r* = 0.661; *p* < 0.001).

Table 5 shows the differences between the two groups in ADL and categorization skills, after controlling for the adjustment variables (age, educational level, socioeconomical status) in the multivariate models (binary logistic regression). Only differences in ROCUS remained after the multivariate analysis (*p* = 0.006). Thus, in our sample, the group of patients with cognitive impairment were more likely to present deficits in categorization skills with unstructured information than the group without cognitive impairment (adjusted OR: 58.31; CI 95%: 3.24–1049.43). According to the interpretation of the OR based on a transformation to Cohen’s d (*d*) [31], described in data analysis subsection, this adjusted OR found in our sample corresponded with a large effect size.

## 4. Discussion

In this preliminary study, we compared the cognitive performance in a categorization task, ROC-d and in ADL of two groups of elderly, with and without cognitive impairment, using ROC-d. In general, it is recognized that poorer functional performance in IADLs is associated with worse cognitive functioning [32]. Deepening the knowledge of which cognitive processes are involved in the performance of IADLs will allow clinicians and researchers to better understand changes in personal independence in older people with cognitive impairment [32].

Our results confirm partially the first hypothesis. Although we found significant differences between the two groups regarding performance of ADLs and categorization skills in bivariate analysis, these differences were maintained only in the categorization of unstructured information (ROCUS), when it was adjusted by age, educational level, and socioeconomic level. Cognitive processes allow us to learn new knowledge and use it in daily life, which requires different mental processes, such as reasoning and categorization skills [33]. Previous studies suggest that cognitive impairment can be detected in neuropsychological tests prior to impaired daily function [3]. Other researchers have indicated that difficulties in IADLs can predict worse cognitive functioning [32,34]; however it is advisable to note that they refer to subjective complaints in the performance of ADLs, indicated by the patient himself [34]. It has been indicated that when the assessment of IADL is conducted through key informants (family members or clinicians) it is possible to accurately distinguish the levels of functional independence, whereas self-report measures do not allow it [32]. A strong point of our study is that the assessment of the ADLs was made through OT who know exactly the functional level of each patient.

The results of the bivariate analysis, regarding the level of independence in the different IADL, support the previous results of other authors. Thus, according to Feger et al. (2020), the difficulties for the performance of the IADL from the age of 75, ordered according to their probability is: travelling, housework, preparing menus, managing finances, managing health, using telephones, and shopping [2]. Moreover, cognitive impairment has been associated with loss of memory, language, and initial reasoning ability in the mild cognitive impairment (MCI) with decreased or abandoned IADLs, such as keeping appointments, using the telephone, and shopping. In cases of moderate impairment, difficulties are also observed in traveling alone, using appliances, finding objects (belongings), selecting clothes, dressing, grooming, maintaining hobbies, and disposing garbage (keeping the environment clean). While in cases of severe dementia, difficulties are already observed in cleaning the table and picking up after eating, walking, being the activity that later lost the food [11]. These results might be discussed alongside the findings of previous studies on neuroimaging, in which it has been observed that the prefrontal areas are the first brain regions to be affected [9]. In fact, many neuropsychological models propose that this prefrontal deterioration plays a fundamental role in the cognitive changes that occur with age [2,35]. This has been explained by the hypothesis of aging of the frontal lobe [9], a region related to cognitive processes and responsible for visuospatial skills and conceptual reasoning [12]. Furthermore, it has been considered that executive skills could account for 54% of the variability in an IADL measure based on the performance of older adults [32].

Regarding the second hypothesis, the results showed a moderate correlation between ROC scores and physical ADLs in the group of people with cognitive impairment. Our results are similar to studies that examined categorization skills using the Wisconsin Card Sorting Test (WCST) to predetermine performance on the Routine Tasks Inventory (RTI-2) among people with severe mental disorders [29]. Although there are studies that suggest that categorization skills are less essential in ADL than other cognitive processes in the population with MCI, such as attention, processing speed, and delayed memory [36], other studies consider them to be more relevant in people with awareness of dementia (perception of abilities) regarding the safety of consciousness, understanding, problem-solving, sequencing, reasoning, and abstract thinking [37]. Also, our results support those of other studies that showed moderate to strong correlations between IADL and executive measures, being capable of accounting for approximately 12–55% of the variability in IADL [32].

On one hand, the present study tries to address a topic in which there are few empirical studies on everyday cognition, trying to determine the cognitive correlates of functional performance, integrating both aspects to improve assessment and develop better treatment plans [37]. Our results provide support to occupational therapy practices focused on the adaptation of the environment, its simplification, in order to facilitate a sense of control of the environment and security for patients with cognitive impairment [38,39,40]. In this way, providing them with structured and ordered information could avoid the confusion, agitation, and other behavioral and emotional problems that are observed in some patients with dementia when performing IADL.

On the other hand, given the interest in considering both quantitative and qualitative aspects during the assessment, an aspect to highlight of the ROC-d is that it allows us to record the patient’s performance and observe carefully if the previous preparatory movements are repeated, if there are perseverations, the order of the cards, the errors made (omission, commission, etc.) and in the analysis of the performance of the task. Likewise, another aspect to point out is that the novelty of the digital test presented, which although similar to the original test, may be attractive when it comes to motivating the patient in the assessment. Alternatively, the tool offers users the possibility to recreate the execution of the patient’s session, which allows to obtain qualitative information on how the task is executed, the type of errors that it commits, and it keeps the order in which they have been performed. This aspect is especially relevant in patients with cognitive deficits and brain injury.

A limitation that some cognitive tests present in people with cognitive impairment or dementia are the ceiling and floor effect in MCI and mild Alzheimer’s disease (AD), which limits their sensitivity to changes over time [41]. Instruments that assess the performance of IADL can complement cognitive assessments by revealing whether a clinically significant change has occurred [42]. Therefore, the combination of sensitive cognitive and functional tests can provide a useful tool to detect clinically relevant changes over time in MCI and dementia.

Finally, our results suggest that the decline in daily life activities cannot be explained exclusively by cognitive processes. Thus, the results seem to indicate that an older age itself, a lower educational level and a lower socioeconomic level could explain lower participation in ADL. This supports a broad and multifactorial conceptualization of participation in the different activities of daily living [43], where although the underlying cognitive processes are important, they are not the only factors (i.e., physical, social, cultural environment), as proposed by the model of functioning, disability and health from WHO [44] or the conceptual models of occupational therapy [45,46].

This study has several limitations that should be considered in interpreting the results. First, an important limitation is the reduced sample size, so the conclusions derived from it must be interpreted with caution. It is advisable to replicate the study with larger samples that allow comparison between different levels of mild and moderate cognitive impairment. Second, selection of participants was done using non-probabilistic convenience sampling. This may limit the extrapolation of the results, although the usefulness of this method in exploratory studies such as ours has been demonstrated [47]. Third, no other patient health status variables were collected during the assessment, so it has not been possible to analyze whether other health variables could be potential confounders. Future studies should collect these variables.

## 5. Conclusions

To the knowledge of the authors, this is the first study on ADL and categorization skills in older people. This study showed that people with cognitive impairment perform poorly in categorization task with unstructured information. The results suggest a high correlation between cognitive functioning and the performance of ADLs. Furthermore, there is a moderate correlation between ADLs and categorization skills in older people.

The ROC-d allows the assessment of categorization skills to be quick and easy, facilitating the assessment process by OT, as well as the accuracy of the data obtained.

## Figures and Tables

**Table 1:** Demographic characteristics of the sample (*n* = 51).

Characteristics	*n*	%
Gender		
Male	16	31.4
Female	35	68.6
Educational Level		
Illiterate	14	27.5
Primary education	16	31.4
Secondary education	14	27.5
Higher education	7	13.7
Socioeconomic status		
Level I	8	15.7
Level II	3	5.9
Level III	40	78.4

Level I: Directors, managers and professionals with university training; Level II: intermediate occupations and self-employed; Level III: Manual workers.

**Table 2:** ROC-d assessment score.

ROCUS	
Score	Criteria
1	Exact identification
2	Follows an incomplete criterion
3	Follows a criterion with spatial preparation
4	Follows a criterion with random disposition
5	Follows two or more criteria simultaneously
**ROC**	
**Score**	**Criteria**
1	Exact identification
2	Follows an incomplete criterion
3	Follows a criterion
4	Follows two or more criteria simultaneously only
	at the second attempt
5	Follows two or more criteria simultaneously at the
	first attempt

**Table 3:** Characteristics of the sample and differences between the two groups of comparison.

	No Cognitive Impairment (*n* = 20)		Cognitive Impairment (*n* = 31)		*p*-Value
	% (*n*)		% (*n*)		
Gender					0.360 *
Male	40 (8)		25.8 (8)		
Female	60 (12)		74.2 (23)		
Educational Level					<0.001 **
Illiterate	0 (0)		45.2 (14)		
Primary education	30 (6)		31.4 (16)		
Secondary education	35 (7)		27.5 (14)		
Higher education	35 (7)		13.7 (7)		
Socioeconomic status					0.004 *
Level I	35 (7)		3.2 (1)		
Level II	10 (2)		3.2 (1)		
Level III	55 (11)		93.5 (29)		
	**Mean (SD)**	**Median (25–75% interquartile range)**	**Mean (SD)**	**Median (25–75% interquartile range)**	
Age	62.40 (5.51)	62 (58.50–64.50)	76.97 (10.66)	81 (64–86)	<0.001 ***
MoCA Score	27.20 (1.20)	27 (26–28)	15.84 (6.05)	16 (10–22)	<0.001 ***
ROC	4.55 (0.95)	5 (5–5)	2.97 (1.02)	3 (2–3)	<0.001 ***
ROCUS	4.95 (0.22)	5(5–5)	3.10 (1.14)	3 (2–4)	<0.001 ***
ROC-d (s)	501.05 (161.30)	484 (366–610)	667.76 (338.42)	642 (327–909)	0.150 ***
Physical ADLs-RTI-2	5.13 (0.04)	5.14 (5.14–5.14)	4.35 (0.77)	4.43 (3.86–5)	<0.001 ***
Grooming	5 (0)	5 (5–5)	4.32 (0.83)	5 (4–5)	<0.001 ***
Dressing	5 (0)	5 (5–5)	4.39 (0.96)	5 (4–5)	0.002 ***
Bathing	5 (0)	5 (5–5)	4.16 (1.19)	5 (4–5)	<0.001 ***
Walking/Exercising	4.90 (0.31)	5 (5–5)	4.06 (1.12)	4 (4–5)	0.001 ***
Eating	5 (0)	5 (5–5)	4.45 (0.57)	4 (4–5)	<0.001 ***
Toileting	5 (0)	5 (5–5)	4.68 (0.48)	5 (4–5)	0.005 ***
Taking Medication	6 (0)	6 (6–6)	4.35 (1.31)	4 (3–6)	<0.001 ***
IADLs-RTI-2	5.31 (0.78)	5.64 (4.93–5.86)	3.42 (1.22)	2.86 (2.43–4.29)	<0.001 ***
Housekeeping	4.30 (1.38)	5 (4.25–5)	2.81(1.66)	3 (1–5)	0.002 ***
Preparing and securing food	5.10 (1.65)	6 (4.50–6)	3.23 (1.67)	2 (2–5)	0.001 ***
Spending Money	5.35 (1.35)	6 (6–6)	3.61(1.52)	3 (3–6)	<0.001 ***
Shopping	5.80 (0.89)	6 (6–6)	3.26 (1.53)	2 (2–4)	<0.001 ***
Doing Laundry	5.25 (1.25)	6 (4.25–6)	3.77 (1.23)	3 (3–5)	<0.001 ***
Traveling	5.45 (0.95)	6 (5–6)	3.32 (1.40)	3 (2–4)	<0.001 ***
Telephoning	5.95 (0.22)	6 (6–6)	3.97 (1.45)	3 (3–6)	<0.001 ***

Level I: Directors, managers and professionals with university training; Level II: intermediate occupations and self-employed; Level III: Manual workers; *: Chi-square test; **: Fisher’s exact test; ***: Mann–Whitney test.

**Table 4:** Spearman correlation between ADL and categorization skills.

	IADL-RTI-2	Physical ADL-RTI-2	MoCA	ROCUS
Physical ADL-RTI-2	0.818 **			
MoCA	0.740 **	0.798 **		
ROCUS	0.510 **	0.547 **	0.732 **	
ROC	0.463 **	0.586 **	0.653 **	0.661 **

** *p* < 0.001.

**Table 5:** Multivariate model with binary logistic regression for ADL and categorization skills.

Score		No Cognitive Impairment *n*%		Cognitive Impairment *n*%		*p*-Value Crude Model	*p*-Value Adjusted for Age, Educational Level and Socioeconomic Status	OR Adjusted for Age, Educational Level, and Socioeconomic Status (CI 95%)
Physical ADL-RTI-2	≤5	2	10.0%	25	80.6%	<0.001	0.179	4.25 (0.52–35.08)
	>5	18	90.0%	6	19.4%			
IADL-RTI-2	≤4.3	3	15.0%	25	80.6%	<0.001	0.998	2.27 × 10^13^ (0–∞)
	>4.3	17	85.0%	6	19.4%			
ROCUS	2–4	1	5.0%	26	83.9%	<0.001	0.006	58.31 (3.24–1049.43)
	5	19	95.0%	5	16.1%			
ROC	1–3	4	20.0%	25	80.6%	<0.001	0.139	4.14 (0.63–27.15)
	4–5	16	80.0%	6	19.4%			

## Data Availability

The data presented in this study are available on request from the corresponding author. The data are not publicly available due to privacy reasons.
